# Transcriptome Analysis of Intrusively Growing Flax Fibers Isolated by Laser Microdissection

**DOI:** 10.1038/s41598-018-32869-2

**Published:** 2018-10-01

**Authors:** Tatyana Gorshkova, Tatyana Chernova, Natalia Mokshina, Vladimir Gorshkov, Liudmila Kozlova, Oleg Gorshkov

**Affiliations:** Kazan Institute of Biochemistry and Biophysics, Federal Research Center “Kazan Scientific Center of RAS” 420111, Lobachevsky Str., 2/31, Kazan, Russian Federation

## Abstract

The intrusive growth, a type of plant cell elongation occurring in the depths of plant tissues, is characterized by the invasion of a growing cell between its neighbours due to a higher rate of elongation. In order to reveal the largely unknown molecular mechanisms of intrusive growth, we isolated primary flax phloem fibers specifically at the stage of intrusive growth by laser microdissection. The comparison of the RNA-Seq data from several flax stem parts enabled the characterization of those processes occurring specifically during the fiber intrusive elongation. The revealed molecular players are summarized as those involved in the supply of assimilates and support of turgor pressure, cell wall enlargement and modification, regulation by transcription factors and hormones, and responses to abiotic stress factors. The data obtained in this study provide a solid basis for developing approaches to manipulate fiber intrusive elongation, which is of importance both for plant biology and the yield of fiber crops.

## Introduction

Enlargement of individual cells is a fundamental process of higher plant development. This increase of cell size in a multicellular organism can proceed in three major ways: by symplastic, protrusive, or intrusive growth^1–3^. During symplastic growth, the involved cells increase their surface simultaneously, retaining the contacts between them through the joint middle lamellae and plasmodesmata. Symplastic growth is the most common type, usually characterizing cells in the elongation zones. Protrusive growth is an outgrowth of a cell part; it takes place only in the epidermal layer and leads to the formation of trichomes, such as root hairs, or hairs of the cotton seed (“cotton fibers”). The third type of cell enlargement, the intrusive growth, is a type characterized by a higher rate of a cell enlargement compared with the adjacent cells. During intrusive elongation, a cell squeezes in between the surrounding cells, splitting their middle lamellae. Several types of plant cells can perform the intrusive growth, with fibers being the classical example^2,4–6^. In recent years, the intrusive growth has attracted much attention and has been the subject of discussions in several reviews^3,7–9^, all of which have pointed to the lack of knowledge of the molecular mechanisms underpinning intrusive growth of fibers.

The fiber intrusive elongation is very important for the development of plant architecture as well as for the yield and quality of commercial plant fibers^6,7,10^. Fiber bundles develop within plant organs mainly due to the intrusive elongation of individual fibers^10^. The key processes that a fiber must accomplish during the intrusive growth include enlarging the cell wall surface (via synthesis and secretion of cell wall polymers and loosening of the existing layers) and increasing the vacuole volume (via water uptake, osmolyte accumulation, synthesis of membrane components). Furthermore, an intrusively growing fiber requires conditions that enable it to squeeze in between neighbouring cells—the dissolution of middle lamellae of the cells on the way of invasion and the abolishment of possible wound effects. Characterization of any of these processes in intrusively growing fibers from a molecular point of view remains quite poor. There is not a single paper with the analysis of fibers isolated at this developmental stage by comprehensive high-throughput techniques, like RNA-Seq. Such experiments are highly demanded to approach understanding of the mechanisms of intrusive elongation and its regulation, but have, at most, been performed on complex samples, which together with the intrusively growing fibers contained other cell types at other developmental stages. The quite limited examples of the studies are the description of transcriptome landscapes in sections of the young xylem from aspen wood, which contain xylem vessels and ray parenchyma together with intrusively growing xylem fibers^11^, [cluster e1], or flax stem portion (TOP) that includes intensively elongating fibers together with many other tissues (e.g. epidermis, phloem, parenchyma, cambium, xylem^12^.

To perform the RNA-Seq analysis specifically for intrusively growing fibers, we have chosen primary phloem fibers of flax due to the well-characterized location within the stem and duration of the process^2^. Primary flax phloem fibers originate from the procambium, close to the apical meristem^4^, and at the earliest stages of development they grow symplastically with the surrounding tissues^13^. The intrusive elongation is initiated at a 1–2-mm distance from the stem apex and continues several centimetres down the stem until the so-called “snap point”, where the transition of fibers to cell wall thickening occurs^13,14^. These morphological markers allow the study of stage-specific parameters of fiber development. However, the combination of a significant cell length with only a primary cell wall makes the intrusively growing fibers quite prone to breakage, which hinders their isolation.

To elucidate the molecular events associated with the fiber intrusive elongation, we isolated the intrusively growing fibers through laser microdissection and, for the first time, performed the RNA-Seq analysis for such cells. Comparison of the obtained data with transcriptome landscapes in the tissues from the meristematic stem portions and in fibers at an advanced developmental stage enabled us (1) to characterize the overall picture of the transcriptome in the intrusively growing fibers, and (2) to determine which genes were subjected to up- and down-regulation of transcription at this critical stage of fiber development.

## Results and Discussion

### General transcriptome characteristics of the analyzed samples

**Figure 1:**
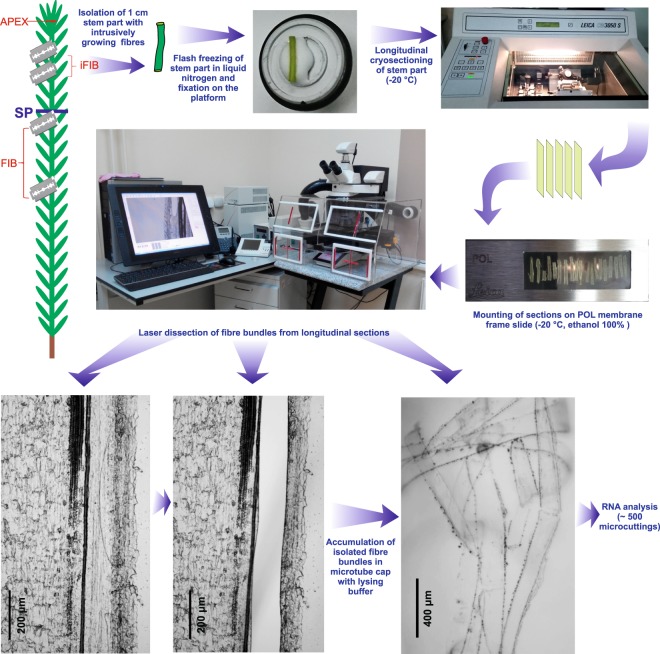
Scheme of plant material collection to obtain APEX, iFIB, FIB samples for subsequent RNA-Seq analysis. Bundles of the intrusively growing fibers (sample iFIB) were isolated by laser microdissection from the longitudinal cryosections of 3rd cm from the stem apex. Sample APEX was collected as 2 mm of the uppermost stem together with leaf primordia. The described earlier^12^ sample FIB (fibers isolated during tertiary cell wall deposition) was collected from the stem portion below the snap point (SP).

To obtain a full picture of the genes expressed during the intrusive growth of fibers *in planta*, we performed a comparative RNA-Seq analysis with the mRNAs isolated from the apical part of the stem (APEX) and the intrusively growing phloem fibers (iFIB) (Fig. 1).

A total of 104,827,884 reads with quality scores above Q30 were acquired; of these,  approx. 90% (92,277,439) were successfully mapped onto the flax reference genome. Normalized expression values of genes were estimated as the fragments per kilobase of exon per million mapped fragments (FPKM). Of the 43,486 protein-coding genes predicted in the whole-genome assembly of flax, 28,983 were found with FPKM >1 (see Supplementary Table S1). FPKM values for the genes in APEX sample were in good agreement (Pearson correlation coefficient for the log_2_ values was r = 0.87) with the data reported by Zhang and Deyholos^15^ for a similar sample verifying our obtained results. The analysis in the software package Cufflinks revealed 10,373 genes with FPKM >16 which could be used for further analysis, as recommended^16^. Using the fold change below 0.33 (down-regulated) and above 3 (up-regulated) as cut-offs for iFIB *versus* APEX, and FPKM >16 at least in one sample, a total of 3,731 differentially expressed genes (DEGs) were identified.

**Figure 2:**
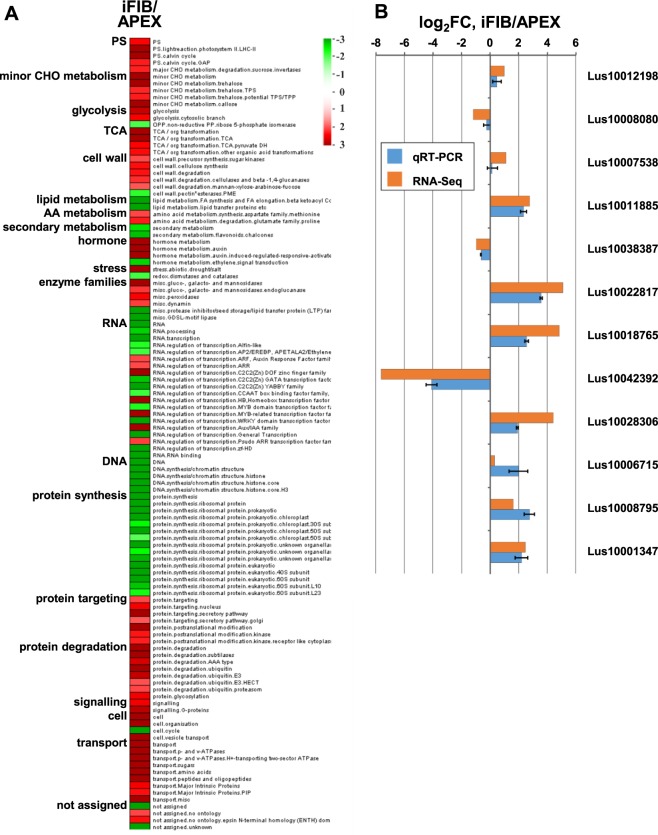
(**A**) Overrepresentation analysis of up- and down-regulated genes within functional gene classes defined by MapMan bins (MapMan, v3.6.0RC1, available online at http://mapman.gabipd.org/web/guest/mapman [accessed on 29 June 2018]) in iFIB sample as compared to APEX (iFIB/APEX). The data were subjected to a Wilcoxon test; resulting p-values were adjusted according to Benjamini and Hochberg in PageMan (http://mapman.gabipd.org/pageman [accessed on 20 July 2018]), and the results are displayed in false color. Functional gene classes colored in red are significantly up-regulated, whereas ones colored in green are significantly down-regulated. PS, photosynthesis; TCA, tricarboxylic acid cycle; AA, amino acid; CHO, carbohydrate. (**B**) Verification of RNA-Seq expression through qRT-PCR, error bar shows the standard error of the mean.

To visualize the changes in gene expression upon transition to the intrusive growth, we compared the transcriptome of iFIB with that of APEX in MapMan^17^, supplementing it with the results of the search of functional classes that were either over- or underrepresented in DEGs between the APEX and iFIB (Fig. 2A) using overrepresentation analysis by the PageMan software^18^. Major metabolic pathways such as photosynthesis, carbohydrate metabolism, cell wall, hormone metabolism, stress, enzyme families, regulation of transcription, protein degradation, signaling, cell organization and transport were overrepresented among expressed iFIB genes. The highest proportion of expressed genes occurred in the pathways designated as lipid and secondary metabolism, regulation of transcription, DNA and protein synthesis, cell cycle.

Twelve genes that had various dynamics of expression between APEX and iFIB were selected for verification by the qRT-PCR. The expression values calculated in RNA-Seq experiments were in good agreement with the data obtained by qRT-PCR (Fig. 2B).

To discriminate the genes that are specifically up- or down-regulated during the intrusive elongation, and those genes that had similar transcript abundance at a later stage of fiber development, we compared our data with the published results of transcriptome analysis for fibers isolated at the stage of the tertiary cell wall formation from 10 cm stem portions taken below the snap point (FIB)^12^ (Fig. 1). Many of the up-regulated differentially expressed genes revealed by comparison of APEX and iFIB samples had similar FPKM values in iFIB and FIB, suggesting that while their expression is important for fiber development, they are not stage-specific. A gene was considered as specifically up- or down-regulated in iFIB, if the fold change in transcript abundance exceeded 3 (|log_2_FC| > 1.6), in comparison with both APEX and FIB. With such a cut-off, 242 genes were up-regulated, while 1151 were down-regulated stage-specifically in iFIB (see Supplementary Table S2). Next, we present the obtained data considering the most important aspects of cell physiology in the intrusively growing fibers.

### Supply of assimilates and support of turgor pressure

**Figure 3:**
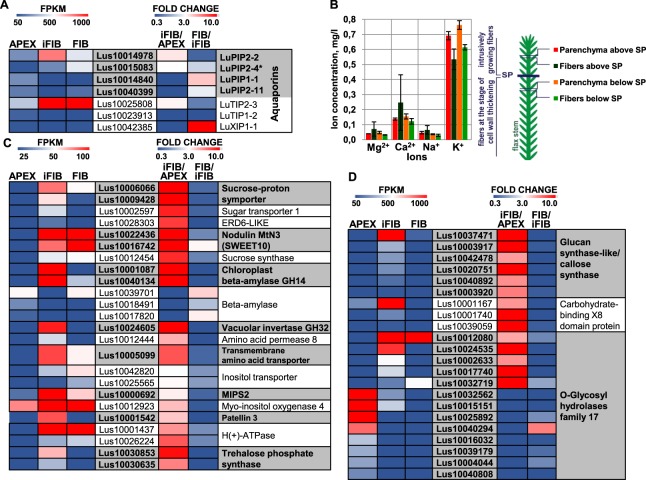
(**A**) Transcriptional level of the differentially expressed in APEX and iFIB genes for aquaporins (PIPs and TIPs, annotated according to^24^ and Arabidopsis homology (*)). (**B**) The concentration of the ions Mg2+, Ca2+, Na+, and K+ in the cortex parenchyma cells and in the fibers above and below the snap point (SP). Ion concentration is expressed as per unit of the total microdissection area. Transcriptional level of the differentially expressed in APEX and iFIB genes for other proteins potentially involved in supplying assimilates, supporting turgor pressure, ion redistribution (**C**) and callose metabolism (**D**). The level of expression (FPKM) is shown on the left side of each panel, while the fold change between the samples is on the right side. The used cut-off was FPKM > 25 (in iFIB or in APEX). Only the genes with the fold change >3 (up-regulated) and with the fold change <0.33 (down-regulated) for iFIB/APEX are shown. Data for the fibers at an advanced stage of specialization (FIB) used for the comparison to determine the genes with a stage-specific character of expression were published previously^12^.

In primary phloem flax fibers intrusive growth leads to the increase of cell surface and volume several thousand times. When these fibers initiate in the procambium they are c. 5 µm in width and less than 100 µm in length, while mature fibers are 20–30-µm wide with a modal length of c. 25,000 µm^7,13^. The characteristic feature of plant cell growth is that an enlargement of cell volume is mainly achieved by the increasing the vacuole through the accumulation of osmolytes and an inward water flux^19^. The latter is largely provided by aquaporins, located in both the plasma membrane (PIP) and the tonoplast (TIP). Different members of multigene families encoding aquaporins were expressed in iFIB and APEX; the genes for PIPs (Lus10014978, Lus10015083) and TIP (Lus10025808) were considerably up-regulated in the intrusively growing fibers (Fig. 3A, see also Lus10032283, Lus10024651, Lus10022611, and Lus10021510 in Supplementary Table S1). This is typical for cells that undergo rapid elongation^20^, such as seed trichomes^21^, maize root cells^22^, and cells in the internodes of deepwater rice^23^. Activation of aquaporin expression was also detected in samples with mixtures of different cell types collected from the flax stem part containing the elongating fibers (ref.^12^, sample TOP). Transcripts for NIPs, SIPs, and XIPs were barely detected in the flax fibers (see Supplementary Table S1) as well as in the flax stem tissues in general^24^.

The nature of the osmoticum in the elongating fibers has not been elucidated yet. Ions were strong candidates. However, our transcriptomic analysis did not reveal any significantly up-regulated genes for ion transporters. In the agreement, the content of major ions was similar between the fibers and surrounding parenchyma, same as in fibers at an advanced stage of development (Fig. 3B). All salt overly-sensitive (SOS) genes were neither differentially nor highly expressed in iFIB (see Supplementary Table S1). Together with that, the genes for the three types of the carbohydrate transporters located in the plasma membrane—sucrose-proton symporter 2 (Lus10009428, Lus10006066); SWEET, also named MtN3^25^ (Lus10016742, Lus10022436); and sugar transport protein 1 (Lus10002597)—were up-regulated in the intrusively growing fibers, similarly to the gene Lus10028303 for a putative early-responsive to dehydration likesugar transporter 6 (ERDL6), which is located on the tonoplast^26^. In addition the specific up-regulation of genes Lus10001087 and Lus10040134 for chloroplast beta-amylase that cleaves starch into low-molecular weight sugars, and for vacuolar invertase (glycosyl hydrolases family 32 protein, Lus10024605) that cuts sucrose into two monosaccharides, rising up osmolarity, occurred (Fig. 3C). Elevated abundance of mRNA for sugar transporters, specific beta-amylases and invertase suggests low-molecular carbohydrates as the most probable osmolytes involved in fiber elongation.

The list of genes for transporters specifically up-regulated in iFIB also included amino acid-proton symporter AAP8 (Lus10012444), member of the transmembrane amino acid transporter family (Lus10005099) and inositol-proton symporters (Lus10042820, Lus10025565) involved in myo-inositol release from vacuoles^27^ (Fig. 3C). Activation of inositol metabolism in intrusively growing fibers is supported by the increased transcript abundance of genes for myo-inositol-1-phosphate synthase 2 (MIPS2, Lus10000692)—the enzyme that catalyzes the rate-limiting step in inositol synthesis^28^, and myo-inositol oxygenase 4 (Lus10012923) involved in the biosynthesis of UDP-glucuronic acid. Additionally, Lus10001542 for the protein from Sec14p-like phosphatidylinositol transfer family (Patellin 3) that may be involved in membrane trafficking events and regulation of phosphoinositide signaling^29^ was specifically up-regulated in iFIB (Fig. 3C).

Plant cell elongation is coupled to acidification of the apoplast^19^. In accordance with that, the genes Lus10026224 and Lus10001437 for H(+)-ATPase that serves as a proton pump were up-regulated in intrusively growing fibers (Fig. 3C). However, the abundance of mRNAs for these genes remained similar at further stages of fiber development. Transcription of the genes for vacuolar proton ATPase (Lus10017490, Lus10028796) was activated less substantially, but more specifically for the elongating fibers (see Supplementary Table S1).

Since plasmodesmata in fibers are disrupted during the intrusive elongation^13^, the intercellular transport is possible only through the fiber apoplast. The increased abundance of mRNA for the sucrose/proton importers (Fig. 3C) may indicate the activation of assimilate transport from the apoplast. Assimilates necessary for cell expansion are also supplied by the fibers themselves, which have fully developed chloroplasts^13^. Genes encoding the components of photosynthesis machinery were up-regulated in the intrusively growing fibers when compared with the meristematic region, including genes for the various components of photosystem II (Lus10004895, Lus10001741, Lus10001169, Lus10006593, Lus10006594), of chloroplast ATP synthase complex (Lus10004894, Lus10032827, Lus10009173) and many others (see Supplementary Table S1).

Symplasmic isolation of cells may be caused by callose plug formation at the plasmodesmata^30,31^. Significant up-regulation of genes for a glucan synthase-like protein (GSL) similar to callose synthase (Lus10037471, Lus10003917, Lus10042478, Lus10020751, Lus10040892, Lus10003920) was specifically pronounced in the intrusively growing fibers (Fig. 3D). Symplasmic isolation of fibers may be required to maintain a high turgor pressure, as shown for elongating cotton seed trichomes^32^. Together with the genes for proteins involved in callose biosynthesis, the genes for callose-binding proteins (carbohydrate-binding X8 domain superfamily proteins; Lus10001740, Lus10001167, Lus10039059) were specifically up-regulated at the intrusive elongation stage (Fig. 3D). Such proteins lack a catalytic domain but can bind the chains of β-1,3-linked glucose in either callose or laminarin^33^. Possible involvement of the X8 domain superfamily proteins in symplasmic isolation of the intrusively growing fibers is suggested by the increased callose accumulation and reduced diffusion of green fluorescent protein from cell to cell in *Arabidopsis* lines with an overexpressed gene of a similar protein, called plasmodesmata callose binding (PDCB) proteins^34^. The partial hydrolysis of callose, if necessary, can be provided by the O-glycosyl hydrolases of family 17; transcription of numerous genes for such proteins was finely adjusted in analyzed samples (Fig. 3D). Genes for the proteins of carbohydrate-binding X8 domain superfamily, O-glycosyl hydrolases family 17, and for the vacuolar invertase similar to those identified in flax fibers were up-regulated in the mixed cell-type samples of aspen wood that contained intrusively growing xylem fibers^11^, [cluster e1].

Additionally, genes for putative trehalose-phosphatase/synthase 9 (TPS9) (Lus10030635, Lus10030853) were specifically up-regulated in iFIB sample of flax (Fig. 3B) and assigned to gene cluster e1 upon analysis of aspen wood samples^11^. Trehalose—a non-reducing disaccharide of glucose, and TPS can be involved in cellular morphogenesis^35^. The revealed similarities (together with other ones listed below) in fiber intrusive growth between different plant species strengthen our conclusions.

### Cell wall enlargement and modification

**Figure 4:**
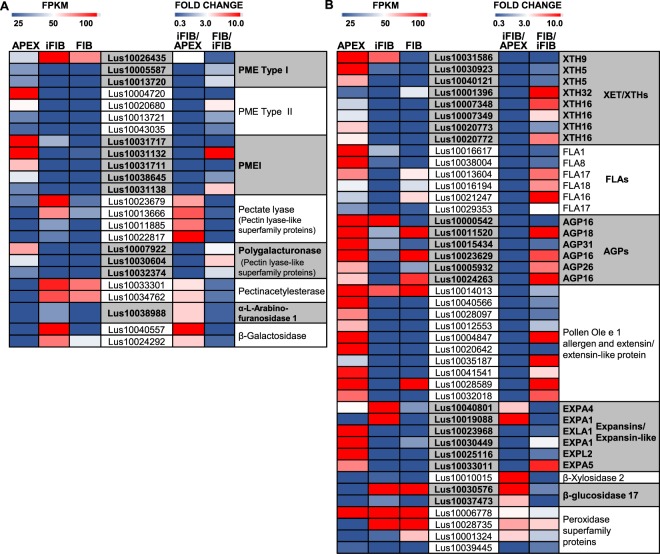
Transcriptional level of the differentially expressed in APEX and iFIB genes encoding cell wall-related proteins. (**A**) Enzymes involved in pectin modification, (**B**) other cell wall enzymes and proteins. The level of expression (FPKM) is shown on the left side of each panel, while the fold change between the samples is on the right side. The used cut-off was FPKM >25 (in iFIB or in APEX). Only the genes with the fold change >3 (up-regulated) and with the fold change <0.33 (down-regulated) for iFIB/APEX are shown. Data for the fibers at an advanced stage of specialization (FIB) used in the comparison to determine the genes with a stage-specific character of expression were published previously^12^.

The primary cell wall of flax phloem fibers is similar to the type I primary cell walls typical for dicots^36^. The major polymers are cellulose, xyloglucan, and pectins. The middle lamella is enriched in pectins, the major constituents of which are homogalacturonan, rhamnogalacturonan I (RG-I), and rhamnogalacturonan II. Among the genes potentially involved in biosynthesis of these non-cellulosic polymers—those encoding corresponding glycosyltransferases and enzymes for interconversion of their monosaccharidic substrates—none were considerably up-regulated in intrusively growing fibers relative to the APEX sample. A moderate up-regulation was detected for several genes of proteins involved in cellulose synthesis machinery, including the CESAs characteristic of the primary cell wall—CESA3 (Lus10007538, Lus10012198) and CESA6 (Lus10041063), cellulase KORRIGAN1 (Lus10007585), and cellulose synthase interactive protein 1 (Lus10019679) (see Supplementary Table S1). At the same time, considerable changes in transcription of genes for the enzymes for the *in muro* modification of the deposited polysaccharides and for other cell wall proteins were pronounced (Fig. 4).

As the intrusive elongation involves splitting of the middle lamella, the corresponding modification of pectins is anticipated. The families of plant enzymes involved in the post-synthetic modification of pectins include polygalacturonases, pectin methylesterases (PMEs) and their negative regulators—pectin methylesterase inhibitor (PMEI) proteins, pectate lyases, pectin acetylesterases, and rhamnogalacturonan lyases. Different sets of genes from large families encoding enzymes for pectin modification were expressed in iFIB and APEX (Fig. 4A).

A stage-specific up-regulation of expression in intrusively elongating fibers was characteristic for representatives of two gene families—those annotated as pectin lyase-like superfamily proteins (Lus10023679, Lus10013666, Lus10011885, Lus10022817) and pectin acetylesterases (Lus10033301, Lus10034762) (Fig. 4A). All the up-regulated in iFIB genes of pectin lyase-like superfamily proteins belonged to the subfamily I of pectate lyases (EC 4.2.2.2, PF00544)—the enzymes that depolymerized demethylated homogalacturonan by transelimination mechanism^37^. Of the numerous genes for polygalacturonases in the flax genome, none was substantially up-regulated in the intrusively growing fibers (see Supplementary Table S1). Out of many tens of PME and PMEI genes^38^, only Lus10026435 had especially abundant mRNA in iFIB as compared to other analyzed samples (Fig. 4A). Herein, several genes for polygalacturonases, PME (both Type I and Type II) and PMEI were down-regulated in iFIB as compared to APEX (Fig. 4A).

Pectin acetylesterases remove acetyl groups that can be present at positions O-2 and/or O-3 of GalA residues in both RG-I and homogalacturonan^39^. The specific up-regulation of genes for such enzymes in the course of fiber intrusive growth points to the importance of pectin’s acetylation degree for this process. This brings up the need for further studies, since currently the biological function of pectin acetylation and the consequences of acetylesterase action are not well understood^39^.

Among the 15 annotated genes for the rhamnogalacturonan lyase protein family, none had an FPKM >16 in the APEX and iFIB samples; herewith, several such genes were highly expressed during tertiary cell wall formation^12^. Modification of RG-I in the intrusively growing fibers is suggested from the stage-specific up-regulation of genes for α-arabinofuranosidase (Lus10038988) and β-galactosidase (Lus10040557, Lus10006009) (Fig. 4B) that may trim off the neutral side chains of RG-I.

The major cross-linking glycan in the primary cell walls of dicots is xyloglucan. Many studies provided evidences favouring the hypothesis of xyloglucan endotransglucosylase/hydrolases (XET/XTH) mediating cell elongation^40–42^. However, gene expression for several XTHs was down-regulated in iFIB, as well as of the genes for fasciclin-like arabinogalactan proteins (FLAs), arabinogalactan proteins (AGPs) and extensin-like proteins (Fig. 4B).

The best-known proteins acting as wall-loosening agents are the expansins^43^. Of the 65 genes encoding expansins and expansin-like proteins present in the flax genome, many had higher expression in APEX than in iFIB. However, some expansin genes (Lus10040801, Lus10019088) were specifically up-regulated in the intrusively growing fibers (Fig. 4B).

In sum, cell wall extension during the intrusive growth is suggested to arise from the action of up-regulated expansins, probable modification of RG-I, and cleavage of homogalacturonan, which occurs by action of pectate lyases rather than of polygalacturonases and may be coupled to deacetylation of the polymer by pectin acetyl esterases.

### Regulation by transcription factors and hormones

**Figure 5:**
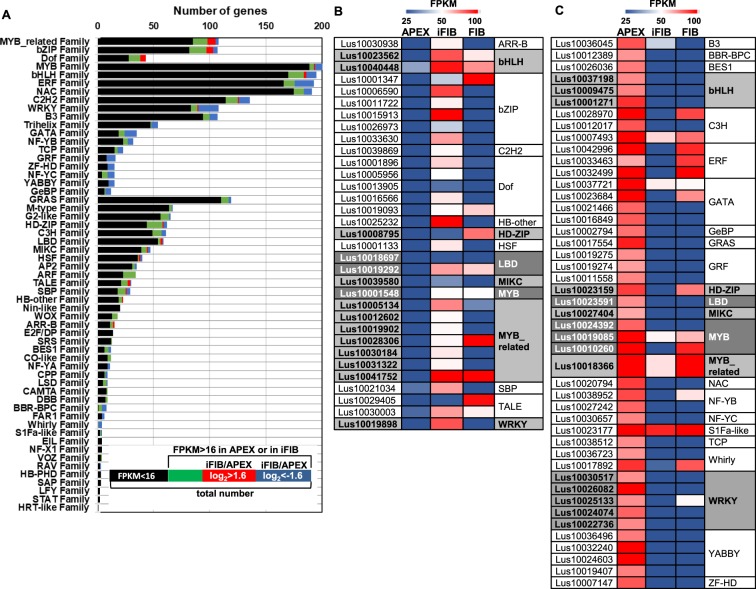
Overview of the transcription factors expressed in APEX, intrusively growing fibers, and fibers at an advanced stage of specialization^12^ (sample FIB). (**A**) The number of genes in the TF families which are presented in the flax genome and those differentially expressed genes in iFIB compared with APEX. Groups of genes that are (**B**) up-regulated and (**C**) down-regulated (using a cut-off FPKM >16 in iFIB or in APEX, |log_2_FC| > 1.6, and FPKM >50 in APEX for down-regulated genes) in iFIB compared with APEX and their expression in fibers forming the tertiary cell wall (FIB).

Key regulatory proteins that play significant roles in many biological processes, by activating and/or repressing transcription, are called transcription factors (TFs). No information about TFs involved specifically in the fiber intrusive growth has yet been obtained. According to PlantTFDB (http://planttfdb.cbi.pku.edu.cn/), the flax genome contains 2481 genes for TFs classified into 57 families (Fig. 5A). Among the up-regulated genes in iFIB relative to APEX, the lists of MYB-related, bZIP and Dof-protein genes were the most extensive, while among the down-regulated genes of TFs, those for the WRKY, AP2/ERF, MYB, GATA, C2H2, bHLH, GRF, NAC, B3, Trihelix, ZF-HD, NF-YC, NF-YB, TCP, YABBY and GeBP families were the most abundant (Fig. 5A). Among the 217 TF genes with a stage-specific character of expression in the intrusively growing fibers, the most represented families were WRKY, C2H2, ERF, MYB, bHLH, GRF, bZIP, Myb-related, NAC and Dof (Fig. 5, see Supplementary Table S2).

The intrusively growing fibers were enriched in transcripts for several genes encoding MYB-related family members. Among those, three genes, Lus10030184, Lus10012602 and Lus10005134, are homologous to At1g01060 (late elongated hypocotyl 1, LHY1), while Lus10031322 and Lus10019902 are homologues of At3g09600 (REVEILLE 8, RVE8) and At2g18330 (RVE7, EPR1). LHY1, RVE8 and RVE7 are known to function as major regulators of the circadian rhythm in plants^44–46^. Up-regulation of these genes in the intrusively growing fibers may thus indicate the importance of the daily cycle in the process of intrusive elongation. In addition, two more up-regulated genes, Lus10028306 and Lus10041752, homologous to At1g75250 (RAD-like 6, RSM3), were identified (Fig. 5B). RAD-like 6 was also up-regulated in the flax^12^ and hemp^47^ stem portions with developing fibers.

The Dof TFs are a group of plant-specific transcription factors that participate in the control of vascular development and functioning^48^. Transcripts of the flax orthologs of AtDof2.3 (Lus10013905, Lus10001896), AtDof3.3 (Lus10019093), and AtDof3.6 (Lus10005956, Lus10016566) were more abundant in iFIB than in APEX (Fig. 5B). AtDof3.6 is supposed to be involved in the control of cell cycle in cambium^48^. AtDof3.6 gene, accumulating in the vascular tissues during the light period, can act as a positive regulator of the phytochrome B signaling pathway and negatively regulate the cryptochrome-mediated seedling photomorphogenesis^48^.

Several families of TFs containing homeodomains, such as HD-ZIP, HB-other, and the closely related TALE, had the up-regulated in iFIB representatives (Fig. 5A,B). Individual members of these families are believed to control the development of the apical meristem and vascular bundles^49^ and to participate in balancing the carbohydrate supply in carbohydrate-consuming tissues^50^. Lus10025232 is a homologue of *Arabidopsis* HomeoBox 1 (AtHB1) that regulates genes involved in cell wall formation and acts downstream of PIF1 (PHYTOCHROME-INTERACTING FACTOR 1) to promote hypocotyl elongation, especially in response to short-day photoperiods^51^.

The transcription factor bZIP34 (Lus10033630), which was found to be specifically up-regulated at the stage of intrusive growth of flax fibers (Fig. 5B), has a close homology to bZIP61 of *Populus* (Potri.019G091900, 56.3%), which was up-regulated in the mixed cell-type samples of aspen wood that contain intrusively growing xylem fibers^11^, [cluster e1]. The expression of these transcription factors was down-regulated in *Arabidopsi*s seedlings at boron deficiency, which is coupled to changes in cell wall properties^52^.

One of the largest families of transcriptional regulators in plants is WRKY, and its members modulate many plant processes^53^. However, these TFs were rather suppressed in the intrusively growing fibers. Only one gene encoding a member of this family (Lus10019898), which is homologous to ATWRKY2, was specifically up-regulated in iFIB (Fig. 5B).

**Figure 6:**
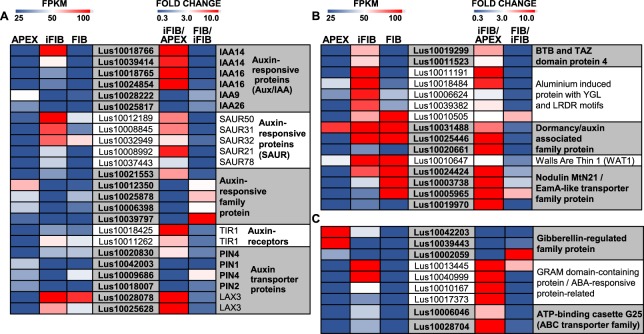
Transcriptional levels of the differentially expressed in APEX and iFIB genes encoding auxin-related (**A**,**B**) and other horomone-related proteins (**C**). The level of expression (FPKM) is shown on the left side of each panel, while the fold change between the samples is on the right side. The used cut-off was FPKM > 25 in iFIB or in APEX. Only the genes with the fold change >3 (up-regulated) and with the fold change <0.33 (down-regulated) for iFIB/APEX are shown. Data for the fibers at an advanced stage of specialization (FIB) used in the comparison to determine the genes with a stage-specific character of expression were published previously^12^.

The regulation of gene expression by TFs is closely interconnected with the action of hormones. Major changes in the expression of hormone-related genes of the intrusively growing fibers occurred with the auxin-related ones (Fig. 6A, see Supplementary Table S2). The list of specifically up-regulated genes contains several genes of auxin-induced proteins that belong to Aux/IAA transcriptional regulators (Fig. 6A). Such proteins do not have a DNA-binding domain, but they do interact with the transcription factors, modulating their activity^54^. Other members of the auxin-signaling pathways with an up-regulated expression in the intrusively growing fibers were small auxin up-regulated RNAs (SAURs) (Fig. 6A). SAURs encode small, plant-specific proteins with no catalytic domains that mediate the effect of auxin in various processes^55^. Notably, the genes for the major receptor of auxin, TIR1 (Lus10011262, Lus10018425), and for auxin signaling F-box 3 (AFB3) protein—other auxin receptor that also mediates Aux/IAA proteins proteasomal degradation and auxin-regulated transcription^56^—were up-regulated in iFIB when compared with APEX (Fig. 6A).

Genes for several auxin transporters—PIN4 (Lus10020830) responsible for the efflux of auxin from a cell, AUX1 (Lus10040212) involved in auxin influx, and auxin transporter-like protein LAX3 (Lus10028078, Lus10025628) that also performs auxin influx—were activated in iFIB when compared with APEX (Fig. 6A and Supplementary Table S2). Genes for calossin-like protein required for polar auxin transport^57^—auxin transport protein BIG (Lus10000087, Lus10005090)—were specifically up-regulated at the stage of fiber intrusive elongation (see Supplementary Table S2). The above-mentioned transporters have different modes of regulation, in combination they provide for the polar transport of auxin^58^. Besides, genes for a plant-specific protein recently identified as vacuolar auxin transporter important for the subcellular distribution of auxin (walls are thin 1, WAT1)^59^ (Lus10010647), and for several WAT1-related proteins previously annotated as nodulin MtN21/EamA-like transporter family protein^25^ (Lus10024424, Lus10003738, Lus10005965, Lus10019970) were up-regulated in iFIB (Fig. 6B).

Genes for two types of proteins that were included by MapMan in the bin of hormonal regulation as auxin induced-regulated-responsive-activated—an aluminum-induced protein with YGL and LRDR motifs, and a dormancy/auxin associated family protein—were highly and specifically expressed in iFIB (Fig. 6B). The same is true for genes (Lus10019299 and Lus10011523) encoding BTB and TAZ domain protein 4 that according to SUBA4 are involved in the response to auxin and gibberellin stimulus. The exact functions of all these proteins have not been elucidated yet.

Other up-regulated genes for hormone-related proteins included four genes (Lus10010167, Lus10010167, Lus10013445, Lus10040999) homologous to the same *Arabidopsis* gene (At5g08350) that encodes the GRAM domain-containing protein from the abscisic acid-responsive protein family, and genes for plasma membrane localized ABC transporter involved in abscisic acid transport and responses (Lus10006046, Lus10028704) (Fig. 6C).

### Reaction to abiotic stress factors

Splitting of the middle lamellae, and squeezing in between neighbouring cells by pushing them aside, would be expected to cause interrelated mechanical, osmotic, and oxidative stresses. As expected, genes of several members of antioxidant and other stress-related systems constituted a large proportion of those genes specifically expressed by the intrusively growing fibers.

**Figure 7:**
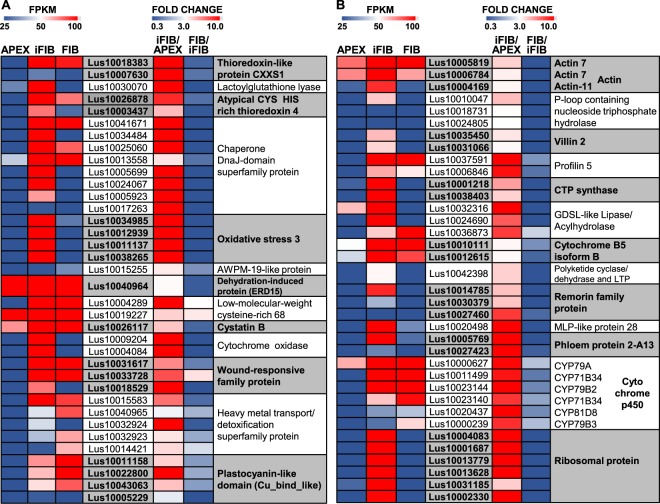
Transcriptional levels of the highly differentially expressed in APEX and iFIB genes encoding proteins with diverse functions. The level of expression (FPKM) is shown on the left side of each panel, while the fold change between the samples is on the right side. A cut-off was used of FPKM >25 (in iFIB or in APEX). The genes with the fold change >3 (i.e., up-regulated) and with the fold change <0.33 (i.e., down-regulated) for iFIB/APEX are shown. Data for the fibers at an advanced stage of specialization (FIB) used in the comparison to determine the genes with a stage-specific character of expression were published previously^12^.

A number of significantly activated genes were those for proteins involved in redox regulation, particularly for several types of thioredoxins (Fig. 7A). Thioredoxins are small oxidoreductases that cleave the disulfide bond between two cysteine residues and are involved in the regulation of the cell redox environment^60^. Lus10007630 and Lus10018383 that were highly activated in iFIB as compared to APEX encoded the specific type of thioredoxin, in which the second cysteine of the redox site was replaced by a serine (thioredoxin-like protein CXXS1); such a monocysteinic thioredoxin has a low disulfide reductase but it has efficient disulfide isomerase activity^61^. None of the genes for thioredoxin reductases, another component of the thioredoxin system, were activated in iFIB (see Supplementary Table S1). Lus10030070 encoding lactoylglutathione lyase/glyoxalase I family protein—the enzyme that uses the reduced glutathione as cofactor for detoxification of methylglyoxal and is associated with stress responses^62^—was highly and specifically activated in iFIB (Fig. 7A); the other member of the same gene family was specifically down-regulated (see Supplementary Table S2).

Many of the proteins encoded by differentially expressed genes are located within the chloroplast, according to the SUBA4 database (http://suba.live/suba-app/factsheet.html)^63^. Transcription of the genes for the chloroplastic atypical CYS HIS rich thioredoxin 4 (Lus10003437 and Lus10026878), which contributes to the fine-tuning of photosynthetic processes^64^, was significantly activated (Fig. 7A). At the same time, genes for the chloroplast-located glutaredoxins (Lus10011915, Lus10013089, and Lus10022844), glutathione peroxidase (Lus10042418), and glutathione S-transferases (Lus10026643, Lus10040347) were specifically down-regulated in iFIB (see Supplementary Table S2), indicating adjustments of redox regulation in the course of full establishment of photosynthetic machinery. mRNAs of genes for the chloroplast-located chaperone, DnaJ, also known as Hsp40 (Lus10025060, Lus10034484, Lus10017263, Lus10024067, Lus10041671), were specifically abundant in iFIB samples (Fig. 7A).

A significant and specific up-regulation was detected for Lus10038265, Lus10011137, Lus10012939, and Lus10034985 (Fig. 7A, see Supplementary Table S2), all of these are homologous to At5g56550, encoding oxidative stress 3 (OXS3) protein, which is known to be involved in plant responses to heavy metals and oxidative stress^65^. Small, (19 kDa) integral membrane protein from AWPM-19-like family was suggested as a suitable molecular marker of osmotic stress and other abiotic stresses^66^. Several genes for members of this plant-specific family (e.g. Lus10001116, Lus10041238, and Lus10011973) were significantly up-regulated in the intrusively growing fibers (Fig. 7A and Supplementary Table S1). The possibility of osmotic stress in the intrusively elongating fibers is also highlighted by the rather specific and considerable up-regulation of genes for an early responsive to dehydration protein 15 (ERD15) (Lus10009847, Lus10040964), much like the genes for members of the rare-cold inducible (RCI) protein family Lus10014028 and Lus10019890 (Fig. 7 and also Supplementary Table S1) which homolog RCI2A was also induced by dehydration and by salt stress^67^. The genes for two types of small cysteine-rich proteins were up-regulated in iFIB: low-molecular-weight cysteine-rich 68 (LCR68) (Lus10004289, Lus10019227) protein that belongs to plant defensins, and cystatin B (Lus10026117)—proteinase inhibitor that can be induced by multiple abiotic stresses^68^ (Fig. 7A).

The detected pronounced changes in the transcription of genes for mitochondrial proteins included the specific up-regulation of Lus10027755 and Lus10035538, which encode the uncoupling mitochondrial protein involved in protecting plant cells against oxidative stress damage. Besides, genes for the members of mitochondrial electron transfer chain—cytochrome c oxidase (Lus10004084 and Lus10009204), and NADH dehydrogenase (Lus10000460, Lus10009720 and Lus10004838)—were highly activated at the stage of fiber intrusive elongation (see Supplementary Tables S1 and S2). Genes for poorly characterized proteins annotated as wound-responsive (Lus10031617, Lus10033728 and Lus10018529), which *Arabidopsis* homologues, according to SUBA4^63^, encode proteins localized in mitochondria, were substantially up-regulated in iFIB (Fig. 7A).

Several genes for the so-called metallochaperones, belonging to the heavy metal transport/detoxification superfamily (e.g. Lus10032924, Lus10015583, and Lus10017730), were also up-regulated in the intrusively growing fibers (Fig. 7A). Cu-binding proteins, which genes (Lus10011158, Lus10022800, Lus10043063, and Lus10005229) were highly activated in iFIB (Fig. 7A), had plastocyanin-like domain and belonged to the family of cupredoxins—small proteins containing type-I copper centres and functioning as electron transfer shuttles between proteins^69^. The significant changes were found in the transcription of genes encoding copper transporters (COPT) COPT1 (Lus10017204) and COPT4 (Lus10021108) (see Supplementary Table S1).

### Other genes specifically expressed in the intrusively growing fibers

Several genes specifically expressed in the intrusively growing fibers encode proteins of the cytoskeleton. Their function in these fibers may be related to the intensive cell elongation and to the development of multinuclearity: phragmoplast is not formed and karyokinesis is not coupled to cytokinesis^13^. Genes for actin 7 (Lus10005819, Lus10006784) and for actin 11 (Lus10004169), same as for beta-6 tubulin (Lus10008528) (Fig. 7B, see Supplementary Table S2) were specifically up-regulated in the intrusively growing fibers; the abundance of corresponding mRNAs diminished at a more advanced stage of fiber specialization. Similar dynamics of expression was observed for genes of the kinesin-like protein FRA1 (Lus10010047, Lus10018731, and Lus10024805) from the P-loop containing nucleoside triphosphate hydrolases superfamily. FRA1 contains the N-terminal microtubule binding motor domain and is proposed to contribute to transportation of Golgi-derived vesicles along cortical microtubules to secrete cell wall material^70^. The specifically up-regulated in iFIB Lus10022839 (see Supplementary Table S2) encodes CLIP-associated protein—a microtubule-associated protein that is involved in both cell division and cell expansion. It likely promotes microtubule stability^71^. Genes Lus10031066 and Lus10035450 for actin-binding protein with high homology to animal villin were also specifically activated in intrusively growing fibers (Fig. 7B). Such proteins are involved in actin filaments bundling in a calcium-dependent manner^72^. Besides, genes of profilin 5 (Lus10006846, Lus10037591) were up-regulated in iFIB as compared to APEX (Fig. 7B). Profilins are considered to be a part of the molecular network that coordinates the actions of microtubules and actin filaments^73^. Interestingly, the genes for the CTP synthase family protein (Lus10038403 and Lus10001218) were specifically up-regulated in iFIB (Fig. 7B), same as in sections of aspen wood that contained intrusively growing xylem fibers^11^, [cluster e1]. Such proteins, independently from their role in biosynthesis of pyrimidine nucleotide, are able to polymerize with formation of filaments, called cytoophidia, that are involved in endoreduplication processes in many living organisms^74^.

Genes for several proteins involved in lipid metabolism were also highly and specifically expressed in iFIB (see Supplementary Table S2). Two of them—Lus10024690, and Lus10032316—are predicted to encode the secreted proteins from the GDSL-like Lipase/Acylhydrolase superfamily. Two other considerably up-regulated genes, Lus10010111 and Lus10012615, are annotated as cytochrome B5 isoform B, which is located in the endoplasmic reticulum and takes part in fatty acid metabolism^75^ (Fig. 7B). Besides, Lus10042398 annotated as polyketide cyclase/dehydrase and lipid transport superfamily protein was specifically up-regulated in iFIB sample; its aspen homolog Potri.004G231000 was assigned to gene cluster that describes the mixed cell-type samples of aspen wood containing intrusively growing xylem fibers^11^, [cluster e1].

A pronounced feature of the transcriptome landscape in the studied intrusively growing fibers was an increased abundance of transcripts for several plant-specific proteins with poorly characterized functions. Together with the mentioned above GRAM domain-containing protein and plant defensin, remorins can be considered as such. They are vascular plant-specific hydrophilic proteins localized in the plasmodesmata areas and in membrane rafts^76^. Remorin was identified as a protein specifically phosphorylated in the presence of oligogalacturonides^77^. Several remorin genes—Lus10027460, Lus10014785, and Lus10030379—were specifically up-regulated in iFIB (Fig. 7B), same as Lus10020498 for major latex protein 28 (MLP28) (Fig. 7B). Additionally, two genes (Lus10027423, Lus10005769) for phloem protein 2 (PP2), described as one of the most abundant and enigmatic proteins in the phloem^78^, were considerably and rather specifically up-regulated in iFIB (Fig. 7B).

The set of genes for cytochromes p450 (CYP) was also highly activated in iFIB as compared to APEX, and kept the similar level of high transcription at later stages of fiber development (Fig. 7B). CYPs comprise the largest enzymatic protein family in plants; various CYPs function most commonly as monooxygenases that use different substrates to provide a wide range of secondary metabolites^79^.

The genes for several ribosomal proteins—the constituents of cytosolic, chloroplastic and mitochondrial ribosomes—were highly specifically up-regulated in intrusively growing fibers (Fig. 7B); many of the genes for ribosomal proteins were down-regulated in iFIB when compared to APEX (see Supplementary Table S2). Changes in the set of ribosomal proteins may influence the translation machinery; however, understating the function of certain ribosomal protein is still rather vague^80^.

### Down-regulated genes in the intrusively growing fibers

**Figure 8:**
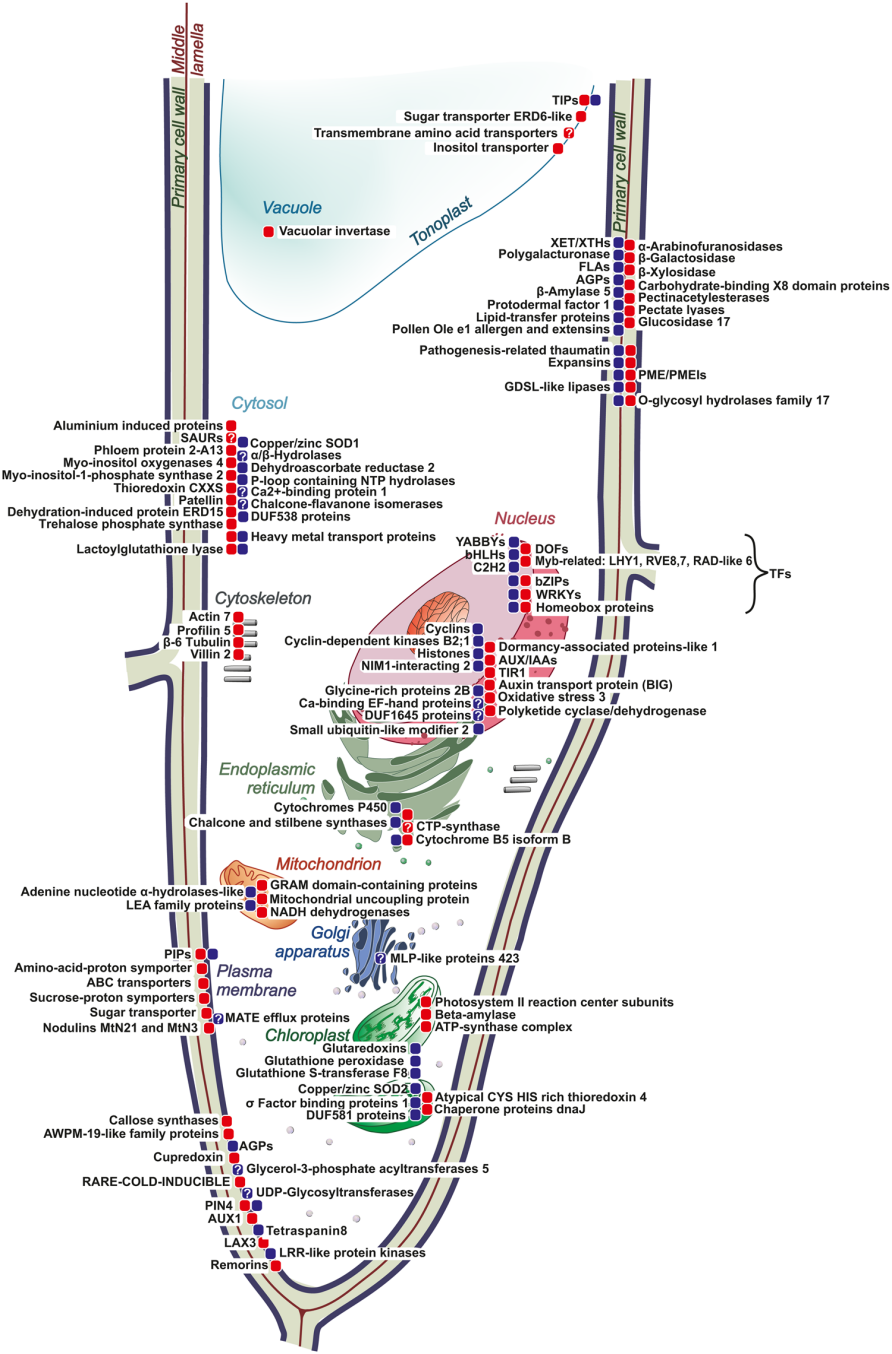
Major molecular players in intrusively elongating flax fiber as revealed by transcriptome analysis. The proteins encoded by genes (gene families) with increased abundance of transcripts are marked by red squares, with decreased abundance—by blue squares. The protein localization corresponds to SUBA4 and UniProt databases. Question sign marks the proteins, which localization is not fully established. Transcript names (according to Phytozome) of genes for presented proteins are listed in Supplementary Table S3.

In total, the expression of 3,145 genes was suppressed in the intrusively growing fibers, when compared with APEX (log_2_FC < −1.6 for iFIB/APEX). The most significantly down-regulated genes included those annotated as cyclins, histones, pathogenesis-related thaumatin, plasmodesmata callose-binding protein 3, as well as some other genes (see Supplementary Table S1). Expression of most of them is the characteristic of apical meristems where they are required for normal cell cycle progression and for meristem organization. Correspondingly, the expression of these genes was detected mainly in the APEX sample: the mRNA abundance of several tens of genes for the histones H2A, H2B, H3, and H4, as well as of 14 genes encoding different classes of cyclins, was reduced in the intrusively growing fibers and was not resumed at the later stages of fiber development. A specific down-regulation at the stage of fiber intrusive elongation was detected for 1,171 genes (log_2_FC < −1.6 for iFIB/APEX and for iFIB/FIB) (see Supplementary Table S2) that belonged to various functional categories. The most important down-regulated genes are presented in Fig. 8.

## Conclusions

The intrusive growth is performed by relatively few cells, which are surrounded by other cells that may grow symplastically, divide, or differentiate, making it very hard to obtain mRNA samples exclusively from the intrusively growing cells. However, by applying a laser microdissection technique, we could progress in filling the knowledge gaps in the molecular mechanisms that rule this peculiar type of plant cell growth. Having in mind that changes in mRNA levels of individual genes are not automatically related to protein synthesis or, especially, enzyme activity, we used the transcriptome profiling as the very indicative approach to realize the patterns of cell metabolism and to identify the key molecular players in the fiber intrusive growth. Based on our obtained data, fiber physiology may be characterized by several major processes that occur simultaneously. Cell elongation goes together with the establishment of active photosynthetic machinery; the latter is coupled to intensive expression of chloroplast-localized chaperonins and thioredoxins. The high proportion of specifically up-regulated genes encodes proteins localized in the cell wall and in the plasma membrane. These include expansins, enzymes for the modification of pectins (pectate-lyases rather than polygalacturonases and glycanases that can cleave off RG-I side chains) and several proteins with poorly characterized functions.

Symplasmic isolation of the fibers within the other stem tissues involves callose and callose-binding proteins, while the intensive water influx to the plant cell is provided by aquaporins (PIPs and TIPs). Our results suggest that low-molecular sugars rather than inorganic ions are the major osmolytes involved in providing turgor essential for fiber growth. The importance of auxin in fiber development is evidenced by the up-regulation of the genes for receptors and transporters of this hormone and for numerous attenuators of its action, such as the SAURs and AUX/IAAs. A specific set of transcription factors is expressed during intrusive fiber elongation. However, to identify the specific roles of each of these transcription factors in inducing and regulating the intrusive elongation *per se*, will require much further research. The most suitable candidates are those TFs revealed in the current study with a stage-specific character of expression.

The major molecular players important for the fiber intrusive elongation as revealed at the transcriptome level are summarized in Fig. 8. Their further characterization would help not only to advance our theoretical understanding of this important aspect of plant biology, but also as tools for breeding for higher yields and quality of fiber crops.

## Materials and Methods

### Plant material and collection of stem samples

Flax plants (*Linum usitatissimum* L., cultivar Mogilevsky from the collection of the All-Russian Flax Research Institute, Torzhok) were grown under open air in boxes with a 50-cm soil layer and received natural daylight and daily watering. Samples for analysis were collected at the fast-growth period of plant development when the stem height was 25–28 cm (30 days after sowing). A set of samples consisted of the manually-cut off apical part of the stem (APEX)—2 mm of the uppermost stem together with leaf primordia—and the intrusively growing phloem fibers (iFIB) that were isolated from 3rd cm from stem apex by laser microdissection from the stem portion above the snap point^14^.

### Cryosectioning and laser microdissection

Figure 1 shows the sequential steps taken to obtain fibers at the stage of the intrusive elongation for the RNA-Seq analysis. Flax stem pieces that had the intrusively growing fibers were cut off from the stem with a razor blade, then immediately frozen in liquid nitrogen and stored at −80 °C. Cryosectioning and isolation of the fiber bundles by laser microdissection followed the protocol of Abbott *et al*.^81^ but modified for flax stem tissues. For cryosectioning, the stem pieces were transferred to a cryostat chamber (CM3050, Leica Microsystems, Wetzlar, Germany) and placed atop a drop of Tissue-Tek O.C.T. Compound (Sakura Finetek Inc., CA, USA). Longitudinal stem cryosections (60-μm thickness) were taken at an object temperature of −20 °C, transferred to a pool of cold 100% ethanol (for the RNA isolation) or deionized water (for the ion content analysis) on a POL-membrane frame slide (Leica Microsystems), and allowed to dry for an hour inside the cryostat chamber at −20 °C.

Longitudinal stem sections were analyzed by using a laser microdissection microscope (LMD7000, Leica Microsystems). Identification of the intrusively growing fibers was based on their localization in the stem (between the xylem and 5–6 cell layers of cortex parenchyma and epidermis), elongated cell shape, and their occurrence in bundles. The microdissections were made at a magnification of 10×, a laser power of 35, and a laser speed of 4; they were collected into the caps of 0.2-ml PCR tubes containing 20 μl of RNA lysis solution (RNAqueous-Micro RNA Isolation Kit, Ambion (Austin, TX, USA)) or deionized water (for the ion content analysis) and stored at −80 °C. The final sample available for the RNA analysis contained about 500 microdissections obtained from 30 plants; for the ion content analysis, it consisted of 30–50 microdissections from 5–7 plants. Ion content was determined in two biological replicates, each in two technical repeats. RNA-Seq analysis was performed for two independent biological replicates.

### RNA extraction and sequencing

Microdissections from the PCR tube with RNA lysis solution were transferred into a microcentrifuge tube with 300 μl of the same lysis buffer for a silica column-based purification. Elution of total RNA was performed with 2 × 10 μl elution buffer preheated to 95 °C. Total RNA from the APEX (20 plants per sample) was isolated using Trizol extraction method combined with RNeasy Mini Kit (Qiagen, Hilden, Germany) according to the manufacturer’s instructions. For all samples, any residual DNA in them was eliminated with a DNA-free kit (Ambion). RNA quantity and quality were analyzed by a Qubit fluorimeter (Invitrogen, Carlsbad, CA, USA) and Agilent 2100 Bioanalyzer (Agilent Technologies). The RNA integrity index (RIN) was more than 6.2, which corresponds to the quality of RNA for further analysis by methods NGS.

For sequencing, total RNA from the APEX (1–3 µg) sample was processed by using TruSeq Sample Prep Kit (Illumina, San Diego, CA, USA) according to the manufacturer’s instruction. To produce normalized cDNA libraries, followed by sequencing using Illumina MiSeq with single-end 75 bp reads. cDNA libraries from total RNA of iFIB samples (up to 1 µg) were prepared with NEBNext Ultra II Directional RNA Library Prep Kit after selective depletion of ribosomal RNA using RiboMinus™ Plant Kit for RNA-Seq according to the manufacturer’s instructions. Sequence was performed on HiSeq2500 with 60 bp single-end reads. Data in the form of raw reads and sample preparation description were deposited in the Sequence Read Archive (SRA) and are available under the Accession Number as the following accessions: Lus_iFIB_1rep: SRR7284881; Lus_iFIB_2rep: SRR7284883; Lus_APEX: SRR7284882; link to BioProject: PRJNA475325 (https://www.ncbi.nlm.nih.gov/bioproject/PRJNA475325).

### RNA-Seq data analysis

The obtained sequence reads (i.e., FASTQ files) were filtered using BBDuk utility of BBMap tools (https://sourceforge.net/projects/bbmap). The clean reads for each sample were mapped onto the flax genome sequence scaffolds^82^ by using HISAT2 v2.1.0^83^ with default parameters using option–dta-cufflinks and then processed by the software Cufflinks^84^. The annotation of flax genes was taken from Phytozome (phytozome.jgi.doe.gov; downloaded file: Lusitatissimum_200_v1.0.readme.txt). Additionally to 43,484 protein-coding genes in the whole-genome assembly of flax^82^ two CESA7 genes^85^ were integrated into the existing annotation and numbered as Lus10043485 and Lus10043486. Normalized expression values of genes were estimated as the fragments per kilobase of exon per million mapped fragments (FPKM), as determined by Cufflinks (v2.2.1) and according to the published protocol^84^. Data from the RNA-Seq analysis were interpreted with the help of MapMan v3.6^17^. and PageMan software^18^ modified for flax plants. To avoid division by zero, a pseudocount of 1 was added when calculating the fold changes (FC). To distinguish the genes that were differentially expressed in the intrusively growing fibers, as compared with more advanced stage of fiber specialization, the data obtained in this study were compared with the previously published results of the transcriptome analysis for fibers isolated during the stage of cell wall thickening^12^ (sample ‘FIB’).

### Validation of transcriptome experiments using qRT–PCR

For quantitative reverse transcription PCR (qRT–PCR), the total RNA (about 0.8 µg) was converted to cDNA with M-MuLV Reverse Transcriptase (Fermentas) using oligo (dT)18 primers according to the standard protocol of manufacturer. Twelve genes identified by RNA-Seq were assayed by qRT–PCR (see Supplementary Table S4) using CFX96 Touch Real-Time PCR Detection System (Bio-Rad). The thermal cycling conditions were 95 °C for 5 min, 40 cycles at 95 °C for 15 s, and 60 °C for1 min. A 60–95 °C melting curve was performed to confirm specificity of the products. From each of two biologically independent cDNA samples two independent technical replications were performed and averaged for further calculations. Relative transcript abundance calculations were performed using the ΔΔCt method^86^ with APEX sample as a reference. The genes encoding basic leucine-zipper 44 (Lus10001347), homeobox 1 (Lus10008795), AGAMOUS-like 19 (Lus10006715), RAD-like 6 (Lus10028306), proline-rich protein 2 (Lus10042392), indoleacetic acid-induced protein (Lus10018765), pectin lyase-like (Lus10011885 and Lus10022817), DUF23 (Lus10038387), DUF642 (Lus10008080), cellulose synthases (Lus10007538 and Lus10012198) were selected for verification by the qRT-PCR. The genes of eukaryotic translation initiation factors 1 A, 5 A (LusETIF1, LusETIF5A) and glyceraldehyde-3-phosphate dehydrogenase (LusGAPDH) were used as the housekeeping genes^87^.

### Ion content analysis

For this analysis, four samples types were collected by microdissection: the intrusively growing fibers, the fibers at the cell wall thickening stage, the cortex parenchyma cells above and below the snap point. Samples were ground by using a glass pestle in deionized water. The qualitative and quantitative analyses of the ions were performed, respectively, by using an ICP-MS ELAN 9000 (Perkin Elmer, Shelton, CT, USA) and an Optima 8300 ICP-OES Spectrometer (Perkin Elmer). Argon flow rate through the outer burner tube: 15 l/min, the middle burner: 0.5 l/min and the sprayer: 0.55 l/min; the power for the plasma operation: 1400 V. The solution flow rate used during the samples’ analysis: 1.5 ml/min; washing time between the samples: 30 s; the time to sample nebulization: 30 s. The samples were analyzed at the following wavelengths: for Mg^2+^: 280.272 nm, Ca^2+^: 396.846 nm, Na^+^: 589.004 nm, and K^+^:766.493 nm. The analysis of these ions was done with two biological replicates; each analyzed twice; the results are conveyed as means ± standard deviation.

## Electronic supplementary material

Supplementary Dataset 1

Supplementary Dataset 2

Supplementary Dataset 3

Supplementary Dataset 4

## Data Availability

All data generated or analysed during this study are included in this published article (and its Supplementary Information files).
